# Hemorrhagic Cystitis With Bladder Tamponade Associated With *Morganella morganii* in an Older Patient Without Recent Catheterization

**DOI:** 10.1002/iju5.70271

**Published:** 2026-09-20

**Authors:** Yoshihiro Kawaguchi

**Affiliations:** ^1^ Department of Urology Morodomi Central Hospital Saga Japan

**Keywords:** aged, hematuria, hemorrhagic cystitis, *Morganella morganii*, urinary tract infections

## Abstract

**Introduction:**

*Morganella morganii* is an uncommon cause of urinary tract infection, typically associated with catheterization or underlying diseases.

**Case Presentation:**

I report a rare case of severe hemorrhagic cystitis with massive intravesical clot retention in a 90‐year‐old woman without recent catheterization or diabetes mellitus. Ultrasonography revealed a large intravesical hematoma requiring clot evacuation. Urine culture yielded *M. morganii* as the sole organism. The patient improved following antimicrobial therapy and supportive management.

**Conclusion:**

This case suggests that *M. morganii* ‐associated urinary tract infection may present with severe hemorrhagic cystitis even in the absence of catheterization. Although the underlying mechanism remains uncertain, urease‐mediated mucosal injury may have contributed to the clinical presentation.

## Keynote Message

*Morganella morganii* urinary tract infection may be associated with severe hemorrhagic cystitis and massive intravesical clot retention even in the absence of catheterization. Urine culture yielded *M. morganii* as the sole organism, and cystoscopy revealed inflammatory bladder mucosal changes without evidence of malignancy. This case highlights the importance of considering *M. morganii* as a potential pathogen in older patients presenting with clinically significant gross hematuria after evaluation for other potential causes.

## 1 Introduction

Urinary tract infections are among the most common bacterial infections and are most frequently caused by *Escherichia coli* and other Enterobacterales [1]. *Morganella morganii* is an uncommon opportunistic pathogen typically associated with underlying diseases, immunocompromised states, or catheter‐related infections [2]. Although generally considered a low‐virulence organism, it can occasionally cause severe and invasive infections [3]. Here, I report a rare case of hemorrhagic cystitis with massive intravesical clot retention associated with *M. morganii* in an older patient without recent urinary catheterization.

## 2 Case Presentation

A 90‐year‐old woman was referred to our department for gross hematuria persisting for several days. She had dementia and was a resident of a nursing facility. She had no history of diabetes mellitus, urinary stones, prior pelvic radiation, previous gross hematuria, or urinary tract infection. Her regular medications were donepezil (5 mg), atorvastatin (10 mg), olmesartan (20 mg), and magnesium oxide (330 mg, three times daily). She was not receiving anticoagulant or antiplatelet medications. Her body temperature was 38.0°C, and assessment of lower urinary tract symptoms was limited by her dementia. Laboratory tests showed a white blood cell count of 7600/μL, a hemoglobin level of 9.3 g/dL, and a C‐reactive protein level of 1.83 mg/dL. The serum creatinine level was 0.63 mg/dL, and the estimated glomerular filtration rate was 65.1 mL/min/1.73 m^2^. Urinalysis demonstrated 2+ leukocytes, 2+ occult blood, and an alkaline urine pH of 8.0; urinary sediment showed 20–29 white blood cells/high‐power field and 50–99 red blood cells/high‐power field.

Blood cultures were not obtained. Before antimicrobial therapy, urine culture and urine cytology specimens were collected by transurethral catheterization. Urine culture yielded *Morganella morganii* (1+, ≥ 10^4^ CFU/mL) as the sole organism, and urine cytology was classified as Class II. Follow‐up urine culture was not performed.

**FIGURE 1:**
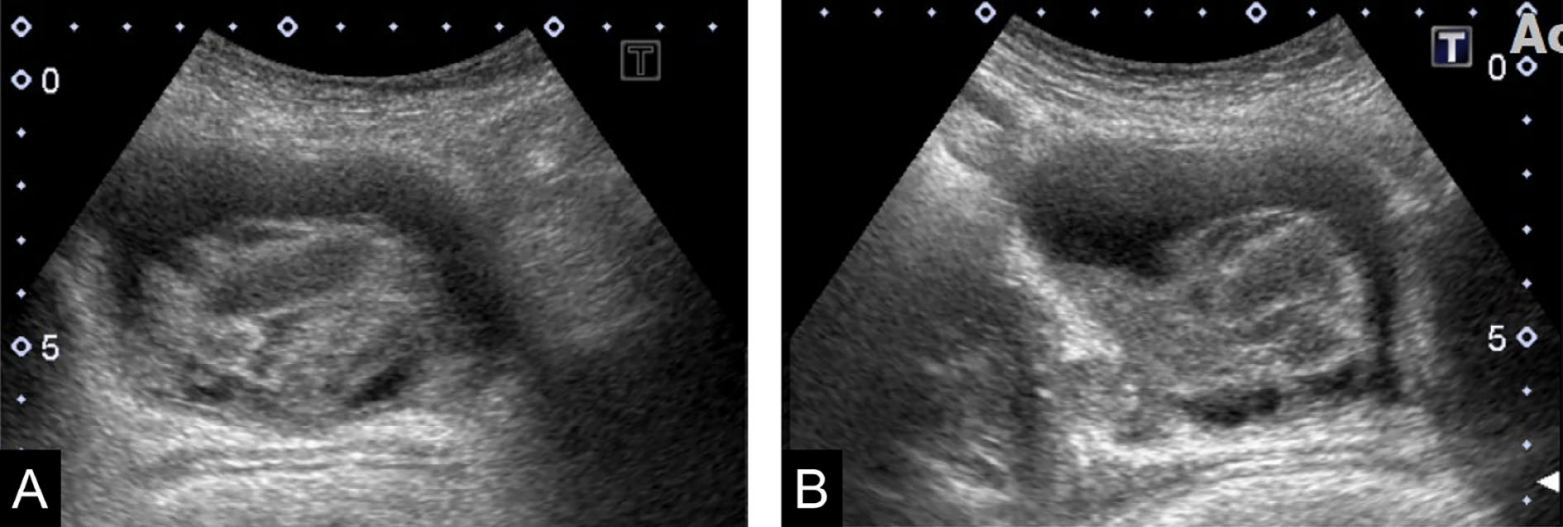
Ultrasonographic findings demonstrating a large intravesical hematoma occupying most of the bladder lumen. (A) Transverse view. (B) Sagittal view.

**FIGURE 2:**
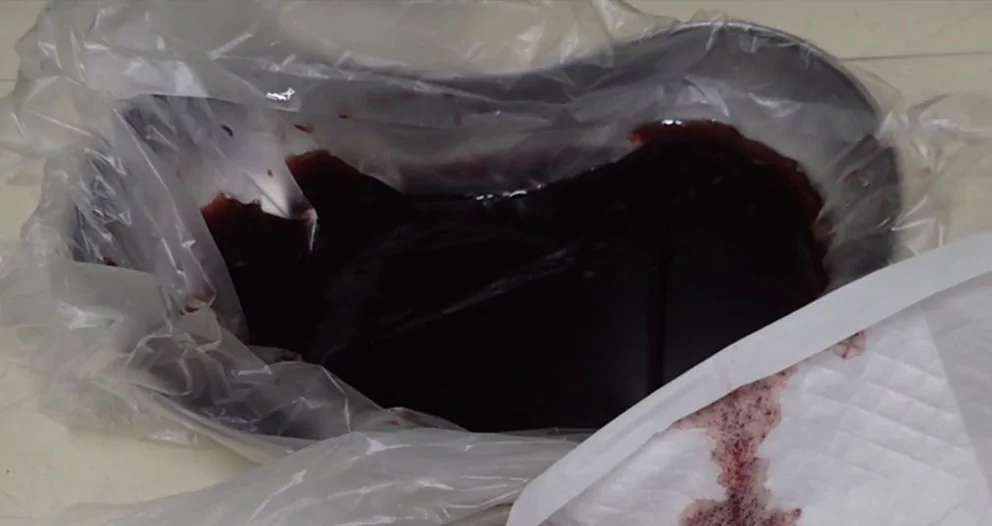
Gross hematuria at presentation. A large amount of clot‐containing urine was evacuated using a metal catheter.

Ultrasonography revealed a large intravesical hematoma occupying most of the bladder lumen (Figure 1). Computed tomography without contrast showed no abnormalities of the bilateral kidneys or ureters, including hydronephrosis or upper urinary tract tumors, and no pre‐existing hematoma was identified. These findings suggested that an upper urinary tract source of hemorrhage was unlikely. Clot evacuation was performed using a metal catheter, followed by urethral catheter placement, and the patient was admitted for further management (Figure 2). Continuous bladder irrigation, blood transfusion, and endoscopic coagulation were not required. No obvious pelvic organ prolapse was observed during the metal catheter procedure. Cefmetazole was initiated, and the patient showed clinical improvement. The urine isolate was susceptible to cefmetazole, which was administered at 1 g every 12 h (2 g/day) for 11 days, followed by oral levofloxacin for 2 days. The urethral catheter was removed on hospital day 7, and once‐daily intermittent catheterization revealed a residual urine volume of less than 100 mL.

**FIGURE 3:**
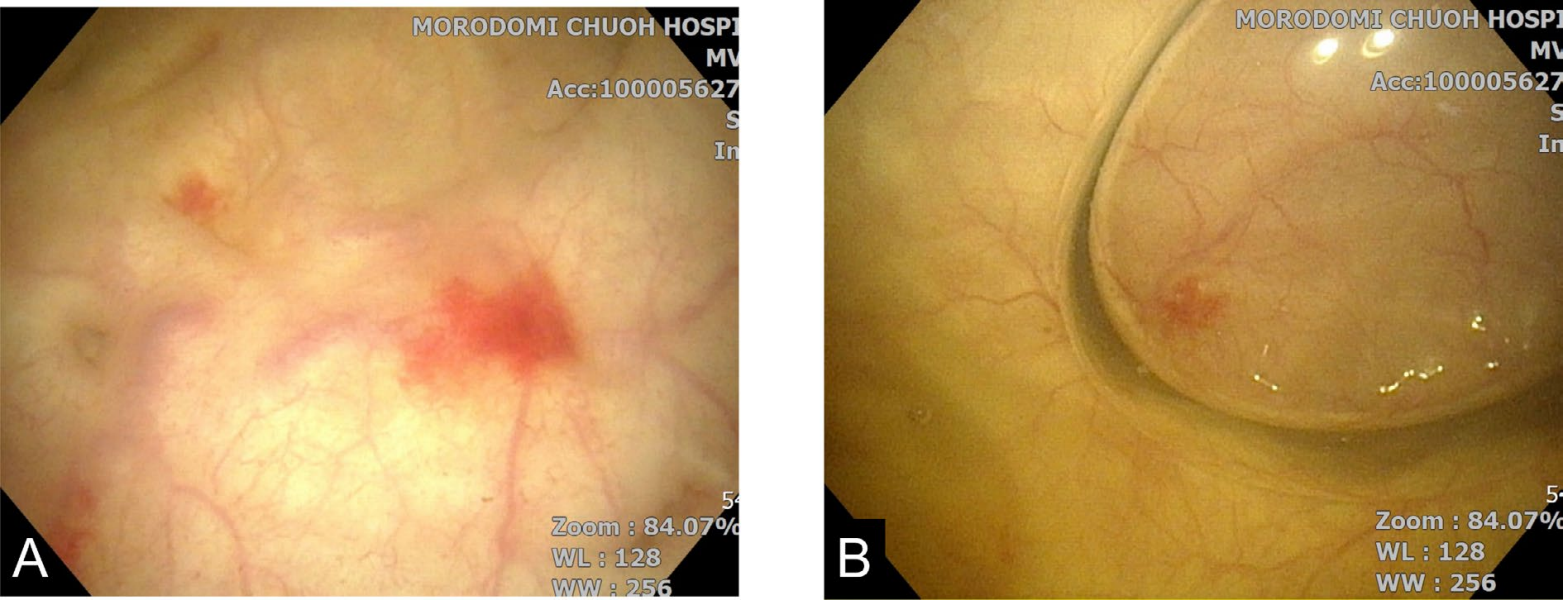
Cystoscopic findings. (A) Prominent erythematous changes are observed on the posterior bladder wall, representing nonspecific inflammatory changes after hemorrhage and instrumentation. (B) Similar erythematous changes are observed on the bladder dome.

**FIGURE 4:**
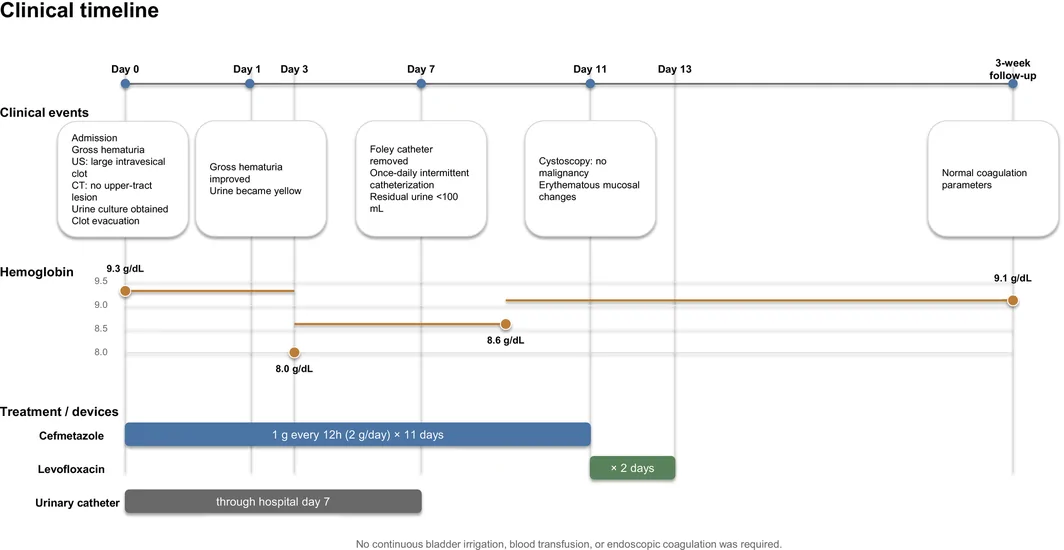
Clinical timeline of the patient's course. The timeline summarizes the clinical course from admission with gross hematuria and bladder tamponade through clot evacuation, antimicrobial treatment, catheter removal, cystoscopy, and follow‐up.

On hospital day 11, cystoscopy revealed no bladder tumor but demonstrated prominent erythematous changes on the posterior bladder wall and dome, suggestive of post‐hemorrhagic inflammatory changes (Figure 3). The hemoglobin level decreased to a minimum of 8.0 g/dL during hospitalization but subsequently remained stable at 8.6 g/dL. At the follow‐up visit 3 weeks after discharge, the hemoglobin level had improved to 9.1 g/dL, and coagulation parameters were within the normal range (Figure 4).

## 3 Discussion

This case provides two important clinical insights. First, *M. morganii* urinary tract infection may occur even in older patients without typical predisposing factors such as diabetes mellitus or recent catheterization [2, 4]. Second, although uncommon, *M. morganii* ‐associated urinary tract infection may present with severe hemorrhagic cystitis accompanied by massive intravesical clot retention and bladder tamponade [2, 5, 6, 7].

*M. morganii* is an uncommon opportunistic pathogen that is typically associated with complicated or catheter‐related urinary tract infections [2, 4, 5]. However, severe invasive infections caused by *M. morganii* have also been reported [3, 6, 7]. In the present case, *M. morganii* was isolated as the sole organism in urine culture in the absence of recent catheterization or diabetes mellitus.

To my knowledge, a PubMed search using the terms “ *Morganella morganii* ,” “hemorrhagic cystitis,” and “gross hematuria” identified no directly comparable reports of *M. morganii* ‐associated hemorrhagic cystitis with massive clot retention and bladder tamponade.

The mechanism underlying the severe hemorrhagic cystitis remains uncertain. I hypothesized that urease produced by *M. morganii* may have contributed to bladder mucosal injury in the present case. Urease hydrolyzes urea into ammonia, which can contribute to urothelial injury [2, 8]. In the present case, CT showed no upper urinary tract abnormalities, and cystoscopy performed after clinical improvement demonstrated erythematous inflammatory changes without evidence of malignancy. Although these findings support a bladder origin of the hemorrhage, they do not establish a causal relationship between *M. morganii* infection and hemorrhagic cystitis. Therefore, the proposed urease‐mediated mechanism should be regarded as a hypothesis rather than an established causal pathway.

This report is limited by its single‐case nature, and the proposed mechanism remains hypothetical because no direct evidence of urease activity, urinary alkalinization, ammonia‐mediated tissue injury, or strain‐specific urease activity was available.

## 4 Conclusion

This case suggests that *M. morganii* ‐associated urinary tract infection may present with severe hemorrhagic cystitis even in the absence of recent urinary catheterization. Clinicians should consider *M. morganii* as a potential pathogen in older patients with clinically significant gross hematuria after exclusion of other causes.

## Funding

The author has nothing to report.

## Ethics Statement

The author has nothing to report.

## Consent

Written informed consent was obtained from the patient for publication of this case report.

## Conflicts of Interest

The author declares no conflicts of interest.

## Data Availability

The data that support the findings of this study are available from the corresponding author upon reasonable request.
